# Global Distribution of Common Variable Immunodeficiency (CVID) in the Light of the UNDP Human Development Index (HDI): A Preliminary Perspective of a Rare Disease

**DOI:** 10.1155/2020/8416124

**Published:** 2020-09-01

**Authors:** Niels Weifenbach, Annalena A. C. Schneckenburger, Stefan Lötters

**Affiliations:** ^1^Private Universität im Fürstentum Liechtenstein, Dorfstrasse 24, 9495 Triesen, Liechtenstein; ^2^Epscheider Straße 6, 58339 Breckerfeld, Germany; ^3^Trier University, Faculty VI Geography / Geosciences, Biogeography Department, Universitätsring 15, 54296 Trier, Germany

## Abstract

Common variable immunodeficiency (CVID), although the most common primary immunodeficiency in humans, is a rare disease. We explored the spatial global distribution and country-wise prevalence of CVID, based on published data and those available from databases. As a country's medical progress is linked to its technological and socio-economic developmental status, we expected that observed CVID prevalence was linked to human wellbeing. To assess this, we examined the correlation of observed CVID prevalence and the UNDP Human Development Index (HDI), which is a key measure of human development. Seventy-four data sets from 47 countries were available (most of them no older than 10 years). Analyses revealed that observed CVID prevalence ranged from 0.001 to 3.374 per 100,000 (mean 0.676 ± 0.83) and was highest in “high” HDI countries (Spearman′s rho = 0.757). Observed prevalence was particularly high in countries where immunodeficiencies are systematically documented in registers. In “low” and “middle” HDI countries, CVID awareness is extremely poor. Assuming that true CVID prevalence does not differ among countries, this study, though preliminary, provides evidence that the discrepancy between observed and (unknown) true prevalence can be clearly linked to the countries' developmental status. As a potential alternative explanation, we briefly discuss the possibility that variation in CVID prevalence is related to human genetic lineage.

## 1. Introduction

Common variable immunodeficiency (CVID) is the most frequently occurring form of primary immunodeficiency in humans. It is characterized by primary hypogammaglobulinemia caused by several different possible factors [1, 2]. Typically, B and T cell abnormalities occur, often only detected relatively late in the life of patients. This immune problem is termed “variable” because its clinical features comprise a wide array of phenomena. Most often, patients suffer from recurrent airway infections. In addition, more serious health issues such as lymphoproliferative autoinflammatory neoplastic disorders, as well as autoimmune diseases (e.g., autoimmune thrombocytopenia), have been reported [1–3].

First described in 1953 and only named “CVID” 20 years later [4], this immune problem is apparently rare [1, 3, 5]. But exactly how rare is it? Systematic documentation has only started in recent years (e.g., [2, 5]), and awareness of the disease among physicians is still considered to be poor, resulting in an unknown number of undiagnosed or wrongly diagnosed patients [1, 6, 7]. Therefore, little robust information is available on prevalence rates, except perhaps for several “industrialized” countries where systematic documentation in register networks has started in recent years [5, 7]. This documentation gives an idea of CVID prevalence, which is suggested to range from <1 to <4 per 100,000 inhabitants (e.g., [8–11]). Selenius et al. [12] even found a rate of 5.5 per 100,000 in Finland, and proposed that variation among countries is the result of slow medical progress. “Emerging” and “developing” countries typically report low prevalence rates at <0.5 per 100,000 inhabitants (e.g., [2, 7, 13–15]). Moreover, for many small “industrialized” countries, no CVID data are available at all (cf. [2, 5, 7]). However, the relationship between development and CVID prevalence remains unclear, as illustrated by the relatively high prevalence rate of Chile (>3 [16]) in contrast with the low rate of the USA (1.5 [7]).

The purpose of this paper is to advance understanding of the spatial global distribution of CVID by country-wise exploring and mapping of CVID. A country's medical progress is linked to its technological and socio-economic status [17]. Considering this, we hypothesize that observed CVID prevalence is positively linked to key measures of human wellbeing.

## 2. Methods

We collected country-wise data (number of cases, year) in two ways. First, in June and July 2019, a literature search in Medline, EMBASE, PubMed, DIMDI, Google Scholar, and Web of Science was performed using “primary immunodeficiency”, “immune deficiency”, “Common Variable Immunodeficiency”, “Common Variable Immune deficiency”, and “CVID” (and/or; all years to present). As the intention of authors was not always to report as much as possible about CVID in their country, we only processed publications dealing with cohorts of N ≥5 CVID cases as a threshold. Kirkpatrick and Riminton [18] considered data for Australia and New Zealand jointly, which, in accordance with Riminton (17 June 2019, pers. comm.), we provisionally corrected for 95% of all cases to be Australian.

Second, access has been granted to the database of ESID (European Society for Immunodeficiencies; https://esid.org/, accessed 16 June 2019) and LASID (Latin American Society for Immunodeficiencies; https://lasid.org/, accessed 24 June 2019). In addition, the freely accessible database of USIDNET, The United States Immunodeficiency Network, was explored (https://usidnet.org/, accessed 15 June 2019). In the absence of an Africa-wide database, ASID (African Society for Immunodeficiencies) was only able to provide CVID data for South Africa (M. Esser, 3 July 2019, pers. comm.). Other primary immunodeficiency registers could not present data on CVID. With regard to the number of CVID cases in databases, no threshold was set for the inclusion of data.

In most of the sources, the number of CVID cases was given for a time period, e.g., 2008–2014 by Marschall et al. [19]. To simplify analyses, we assumed 100% survival of patients at the year when recording terminated and took the maximum accumulated number of known CVID reports to calculate prevalence (number of patients per 100,000 inhabitants [20]) for that year. For this purpose, we used population density data from the World Bank's World Development Indicators database (http://datatopics.worldbank.org/world-development-indicators/, accessed 12 June 2019). Accordingly, for each year, the Human Development Index (HDI; http://hdr.undp.org/en/data; accessed 11 June 2019) was adopted from the annual Human Development Report by the United Nations Development Programme [21, 22]. HDI is a measure of average achievement in key dimensions of human development; it summarizes per capita information on life expectancy, education, and gross national income [23]. The HDI is available for 189 countries. The index ranges from 0 to 1, with countries being classified as having “low” (<0.500), “middle” (0.500–0.799), or “high” (≥0.800) HDI. The measure covers the time period of 1990–2017 or a subset of years within that range. For CVID data from 2018 to 2019, we used the HDI from 2017 because the HDI for 2018 and 2019 had not yet been published.

The correlation between CVID prevalence and HDI was calculated by Spearman's rank correlation coefficient (rho). Statistical analyses were computed in PAST 3.23 [24] and spatial data were processed in DIVA GIS [25].

## 3. Results

### 3.1. Global Distribution and Observed Prevalence of CVID

As shown in Table 1, information from 47 countries from all continents except Antarctica was available for the period 1994–2019. For several countries, information was obtained from different years, so that the total number of data sets was 74. The number of CVID cases spanned an enormous range from 1 in the Dominican Republic (2019) to 4,833 in the US (2019) (median 67). Observed prevalence ranged from 0.001 in India (1994) to 3.374 in Chile (2017) (mean 0.676 ± 0.83). Correcting for the effect of recent attempts to better document primary immunodeficiencies, e.g., by the establishment of national register networks [5], by regarding only the data sets from the last 10 years (*N* = 64), we found the lowest prevalence to be 0.012 in Egypt (2014).

**Figure 1:**
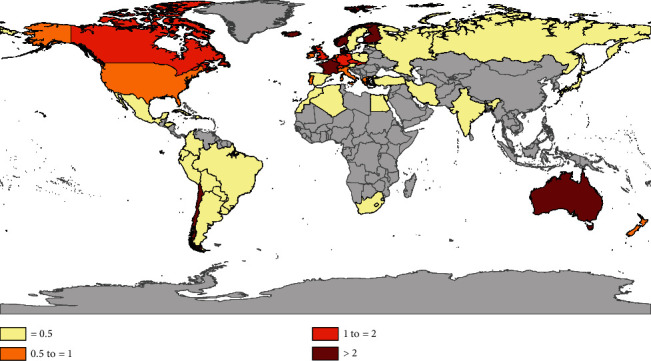
Global distribution of CVID with countries of records shown in color (*N* = 47); observed prevalence is arranged in four classes, based on data in Table 1 (when information from various years was available, the most recent was used). Countries with no CVID records are shown in gray.

Data in Table 1 and Figure 1 demonstrate that we generally know the least about CVID in Africa and Asia. In contrast, observed prevalence is relatively high (from West to East) in North America, Europe, and Australia, where in various countries CVID has been increasingly documented (cf. Table 1). The high prevalence in Chile is remarkable given that comparatively few CVID cases have been reported in other South American countries. Likewise, the relatively low prevalence observed in Sweden stands in sharp contrast to prevalence rates observed in other Nordic countries (Figure 1).

### 3.2. CVID and HDI

**Figure 2:**
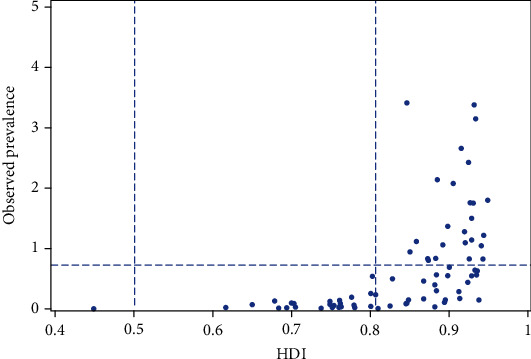
Observed CVID prevalence according to the HDI in 74 data sets (cf. Table 1). The vertical lines mark the cut-off points to classify HDI as “low,” “middle,” and “high.” The horizontal line corresponds to the mean observed prevalence of all data sets.

Among the 74 data sets, the HDI ranged from 0.452 in India (1994) to 0.944 in Switzerland (2019) (mean 0.838 ± 0.095); 51 data sets (68.9%) had a “high,” while 22 had a “middle” and only one had a “low” HDI (Figure 2, Table 1). When accounting for recent improvements in CVID documentation [5] by including only the data sets for the last ten years, and when including only the largest data set per country (*N* = 44), the lower range increased to 0.617 in Honduras (2019). The average remained almost unchanged (mean 0.844 ± 0.084), and there was no country with a “low” HDI.

**Figure 3:**
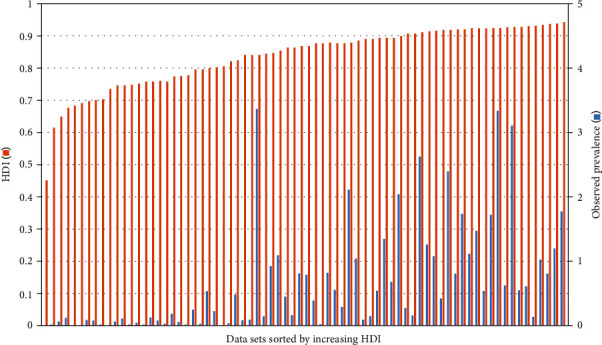
HDI accompanied by CVID prevalence in 74 data sets (cf. Table 1).

Although this average for 44 countries (2009–2019) was only moderately above that of the HDI for all 189 countries in both 2009 (mean 0.677 ± 0.157) and 2017 (mean 0.709 ± 0.152), the difference was highly significant (*p* < 0.001, Mann–Whitney *U* test). This strongly suggests that CVID recognition in general is overrepresented in countries with a higher HDI. It is noteworthy that only the data sets with a “high” HDI—and of these about one half (i.e., 23)—exceeded the mean observed prevalence of CVID (cf. Figure 2, Table 1). In line with these findings, Spearman's rho for observed prevalence and HDI was 0.757. Despite this strong positive linear relationship, prevalence did not necessarily increase with higher HDI (Figure 3). In particular, it is evident that in some countries of high HDI, observed prevalence was markedly below the mean of all data sets, as for instance in Germany (2013, 2014) and Sweden (2014) (Table 1).

## 4. Discussion

### 4.1. CVID Distribution and Prevalence with Regard to HDI

Based on our study, CVID is known from only about one-fourth of the world's countries. This emphasizes that the poor awareness of this disease noted by physicians even in countries where CVID is known (e.g., [1, 7]) is even more drastic at the global scale. While CVID data are mostly recorded in “industrialized” countries, our survey revealed that dramatically little information is available on this disease in Africa and Asia. However, it is noteworthy that there are also highly developed countries for which no information on CVID prevalence is available (to the best of our knowledge), even including some that rank among the “top 25” of highest HDI (e.g., Israel, Singapore [23]).

Among the 47 countries with available CVID records, we hypothesized a positive correlation of observed prevalence and HDI. The latter is a measure of average achievement in key dimensions of human development [23]. We found a strong positive linear relationship supporting this hypothesis. Essentially, 51 of 74 data sets (including multiple years in some countries) originated from “high” HDI countries. In 2017, there were globally 59 “high” HDI countries, suggesting that in general CVID knowledge among those countries is “advanced.” This is a sharp contrast to the 22 data sets from the worldwide 108 “middle” countries and the single data set from one of the 23 “low” HDI countries.

As there is *a priori* no reason to expect that the true incidence differs among countries [1] (but see discussion of alternative explanations below), our findings suggest that in many of the countries where CVID is known, true prevalence should be much higher than observed. Higher true than observed prevalence has already been suggested in earlier CVID studies at smaller spatial scales; these studies suggest that the discrepancy is due to relatively poor CVID awareness among physicians (e.g., [1, 7, 12]). Taking this a step further, our results demonstrate that the discrepancy between observed and (unknown) true prevalence can be clearly linked to countries' technological and socio-economic status. However, given that CVID data were available for fewer than 50 countries, we still regard our results as preliminary, especially as some of the countries with “missing” data also have high HDIs (e.g., Hong Kong, South Korea, Qatar [23]).

### 4.2. The Value of Databases

Over the last one to two decades, our knowledge on CVID has greatly increased (e.g., [2, 3]), and along with new medical centers dedicated to immunodeficiencies, systematic documentation in national or international registers has started in several countries (e.g., [8–11]). The value of such databases [5] is evident in Table 1. In most countries, the number of CVID cases obtained from databases in our study (*N* = 30) was considerably higher than the number of cases for the same country taken from publications, with only a few exceptions, i.e., Chile, the Netherlands, Spain, and the USA. This comparison is not entirely valid, however, as the goal of published studies was not always to count all CVID cases in the respective country. Moreover, in the case of Chile, the published data may overestimate real prevalence, as suggested by Poli et al. [16] themselves, because only ICD-10-coded hospitalizations were used to identify CVID cases.

However, despite these exceptions, our data generally suggest that when CVID data in a country are collected in systematic registers, this gives an “advantage” to those countries with no registers when approaching country-wide prevalence rates. There is a tendency for CVID databases to be predominantly run in “high” HDI countries; two-thirds of all data sets in this study originated from such databases (Table 1). This easily explains why some “high” HDI countries have relatively high observed prevalence rates.

### 4.3. Alternative Explanations

Along with previous authors, e.g., Yong et al. [1], we assume that true CVID incidence does not differ among countries. However, this assumption remains to be tested. The etiology of this immunodeficiency is not fully understood, despite the fact that CVID obviously has a genetic basis and that in the majority of patients, a polygenic cause is likely [26, 27]. Studies so far involve cohorts of some hundred patients from a few countries only (e.g., [26–28]). We do not know whether all people all over the world have equal genotypic preconditions for developing CVID. To date, only Selenius et al. [12] have tentatively discussed whether regionally distinct CVID prevalence rates within Finland could perhaps be explained by influences from genetically distinct founders. Projecting this to the entire world, it cannot be ruled out that distinct genetic lineages (clades) of *Homo sapiens* vary in their potential to develop CVID. That is, the global distribution of CVID and variation in observed prevalence among countries could perhaps alternatively (or additionally) be explained by “race.” Interestingly, according to The United States Immunodeficiency Network (https://usidnet.org/, accessed 15 June 2019), of 1,776 CVID patients, 1,441 (~81%) were described to be “Caucasian.” However, this could also be the result of unequal access to health care among ethnic groups within the country [29].

Although we suggest considering distinct genetic lineages within our species to explain geographic patterns of CVID prevalence, at the current stage, it is premature to use this information as a basis for any concrete hypothesis.

### 4.4. Caveats

Some limitations of this study should be pointed out. About half of our data sets originated from published studies. These publications' aim was not always to provide a country-wide picture of CVID cases. Nevertheless, often these studies were the only available information on CVID cases in a certain country at a certain time. In contrast, as in the Chilean case (see above), data sets may also risk overestimation of prevalence. We are aware that all of these issues create a bias in observed prevalence. However, our goal was to examine the pattern at a large scale rather than make detailed comparisons for particular countries. Moreover, even from certain databases aiming at nation-wide immunodeficiency surveys, the available information can be very limited (cf. Table 1). At the current stage, due to differences in quality of the available data, these problems cannot be solved.

We calculated prevalence using country-wide population data, which is a standard method [20]. This may also lead to bias, as demonstrated by Selenius et al. [12]. These authors calculated CVID prevalence in Finland based on reported cases and population density in districts of hospitals treating CVID; they then used weighted means to extrapolate the prevalence of the entire country. As a result, their country-wide prevalence was higher than that calculated by us (5.5 vs. 2.4). The discrepancy is noteworthy and perhaps gives an idea of the roughness of our data, as the approach of Selenius et al. [12] is certainly more elaborate and exact. However, in our study, CVID cases from most sources could not be allocated to subunits within countries.

Given that some caution must be taken about the completeness of the global data sets and their spatial coarseness, we suggest considering our results as preliminary.

## 5. Conclusions

CVID is a rare disease of globally limited awareness, with an immense lack of knowledge especially in “low” and “middle” HDI countries. Among the countries where CVID has been reported, observed prevalence is positively correlated with increasing HDI. When assuming that true CVID prevalence does not differ among countries, the discrepancy between observed and (unknown) true prevalence can be clearly linked to the countries' developmental status, i.e., HDI. But not all “high” HDI countries have high prevalence rates; rather, these rates were often high in countries where CVID is systematically documented in registers. Also, in future studies, it might be worth considering alternative explanations, such as distinct human lineages and their genotypic preconditions to develop CVID.

## Figures and Tables

**Table 1:** Known CVID cases for 47 countries from various years (74 data sets in total), followed by observed prevalence and HDI. Data sorted in alphabetical order by continent.

Country	Continent	CVID cases	Year	Population density	Observed prevalence	HDI	Source for CVID cases
Algeria	Africa	29	2014	39110000	0.074	0.747	[30]
Egypt	Africa	11	2014	91810000	0.012	0.683	[2]
Morocco	Africa	24	2014	34320000	0.070	0.65	[14]
South Africa	Africa	55	2019	56720000	0.097	0.699	ASID
India	Asia	14	1994	942200000	0.001	0.452	[31]
Iran	Asia	98	2001	76100000	0.129	0.678	[32]
Iran	Asia	208	2019	82360000	0.253	0.798	ESID
Japan	Asia	136	2011	127800000	0.106	0.89	[33]
Australia	Australia	441	2007	20830000	2.117	0.881	[18]
New Zealand	Australia	23	2007	4224000	0.545	0.894	[18]
Austria	Europe	25	2019	8860000	0.282	0.908	ESID
Belgium	Europe	19	2014	11180000	0.170	0.909	[2]
Belgium	Europe	123	2019	11350000	1.084	0.916	ESID
Czechia	Europe	87	2014	10510000	0.828	0.879	[2]
Czechia	Europe	111	2019	10590000	1.048	0.888	ESID
Denmark	Europe	179	2017	5749000	3.114	0.929	[11]
Estonia	Europe	6	2014	1316000	0.456	0.864	[2]
Finland	Europe	132	2017	5503000	2.399	0.92	[12]
France	Europe	532	2005	64610000	0.823	0.869	[9]
France	Europe	252	2008	63960000	0.394	0.878	[34]
France	Europe	894	2014	66130000	1.352	0.894	[2]
France	Europe	1377	2019	66990000	2.056	0.901	ESID
Germany	Europe	512	2013	80770000	0.634	0.928	[2]
Germany	Europe	451	2014	81200000	0.555	0.93	[2]
Germany	Europe	856	2019	82800000	1.034	0.936	ESID
Greece	Europe	18	2014	10930000	0.165	0.864	[2]
Greece	Europe	85	2019	10700000	0.794	0.87	ESID
Iceland	Europe	11	2015	329100	3.342	0.927	[10]
Ireland	Europe	28	2005	4112000	0.681	0.896	[35]
Ireland	Europe	38	2014	4638000	0.819	0.921	[2]
Ireland	Europe	40	2019	4900000	0.816	0.938	ESID
Italy	Europe	20	2016	60670000	0.033	0.878	[3]
Italy	Europe	338	2019	60480000	0.559	0.88	ESID
Netherlands	Europe	190	2014	16830000	1.129	0.924	[2]
Netherlands	Europe	107	2019	17190000	0.622	0.931	ESID
Norway	Europe	117	1999	4450000	2.629	0.911	[36]
Poland	Europe	32	2014	38480000	0.083	0.842	[2]
Portugal	Europe	96	2019	10290000	0.933	0.847	ESID
Slovakia	Europe	8	2014	5416000	0.148	0.845	[2]
Slovakia	Europe	60	2019	5435000	1.104	0.855	ESID
Spain	Europe	213	1995	39852000	0.534	0.8	[8]
Spain	Europe	139	2014	46770000	0.297	0.88	[2]
Spain	Europe	69	2019	46450000	0.149	0.891	ESID
Sweden	Europe	14	2014	9645000	0.145	0.933	[2]
Switzerland	Europe	98	2014	8140000	1.204	0.939	[19]
Switzerland	Europe	152	2019	8542000	1.779	0.944	ESID
UK	Europe	810	2013	64110000	1.263	0.915	[37]
UK	Europe	281	2014	64600000	0.435	0.919	[2]
UK	Europe	1156	2019	66470000	1.739	0.922	ESID
Russian Federation	Europe/Asia	57	2012	143200000	0.040	0.798	[38]
Russian Federation	Europe/Asia	9	2014	143800000	0.006	0.807	[2]
Turkey	Europe/Asia	65	2012	74720000	0.087	0.76	[13]
Turkey	Europe/Asia	15	2014	76670000	0.020	0.778	[2]
Canada	North America	642	2018	37060000	1.732	0.926	[7]
Mexico	North America	43	2014	124200000	0.035	0.761	[39]
USA	North America	4833	2017	325700000	1.484	0.924	[7]
USA	North America	1776	2019	327350000	0.543	0.924	USIDNET
Argentina	South America	21	2016	43850000	0.048	0.822	[40]
Argentina	South America	218	2019	44270000	0.492	0.825	LASID
Bolivia	South America	2	2019	11050000	0.018	0.693	LASID
Brazil	South America	51	2016	207700000	0.025	0.758	[15]
Brazil	South America	291	2019	209300000	0.139	0.759	LASID
Chile	South America	17	2019	18050000	0.094	0.843	LASID
Chile	South America	609	2017	18050000	3.374	0.843	[16]
Colombia	South America	13	2007	44370000	0.029	0.704	[41]
Colombia	South America	60	2019	49070000	0.122	0.747	LASID
Cuba	South America	7	2019	11480000	0.061	0.777	LASID
Dominican Republic	South America	1	2019	11003000	0.009	0.736	LASID
Ecuador	South America	9	2019	16620000	0.054	0.752	LASID
Honduras	South America	2	2019	9265000	0.022	0.617	LASID
Mexico	South America	246	2019	129200000	0.190	0.774	LASID
Paraguay	South America	6	2019	6811000	0.088	0.702	LASID
Peru	South America	7	2019	32170000	0.022	0.75	LASID
Uruguay	South America	8	2019	3457000	0.231	0.804	LASID

## Data Availability

Data is available upon request and may be obtained by contacting the corresponding author.
